# Towards More Accurate and Complete Heterogeneous Iris Segmentation Using a Hybrid Deep Learning Approach

**DOI:** 10.3390/jimaging8090246

**Published:** 2022-09-10

**Authors:** Yuan Meng, Tie Bao

**Affiliations:** College of Computer Science and Technology, Jilin University, Changchun 130012, China

**Keywords:** image segmentation, iris segmentation, semantic segmentation, CNNs, deep learning

## Abstract

Accurate iris segmentation is a crucial preprocessing stage for computer-aided ophthalmic disease diagnosis. The quality of iris images taken under different camera sensors varies greatly, and thus accurate segmentation of heterogeneous iris databases is a huge challenge. At present, network architectures based on convolutional neural networks (CNNs) have been widely applied in iris segmentation tasks. However, due to the limited kernel size of convolution layers, iris segmentation networks based on CNNs cannot learn global and long-term semantic information interactions well, and this will bring challenges to accurately segmenting the iris region. Inspired by the success of vision transformer (VIT) and swin transformer (Swin T), a hybrid deep learning approach is proposed to segment heterogeneous iris images. Specifically, we first proposed a bilateral segmentation backbone network that combines the benefits of Swin T with CNNs. Then, a multiscale feature information extraction module (MFIEM) is proposed to extract multiscale spatial information at a more granular level. Finally, a channel attention mechanism module (CAMM) is used in this paper to enhance the discriminability of the iris region. Experimental results on a multisource heterogeneous iris database show that our network has a significant performance advantage compared with some state-of-the-art (SOTA) iris segmentation networks.

## 1. Introduction

Iris recognition has been widely applied in security, e-commerce, finance, etc. Iris segmentation is when iris regions are segmented from a lot of interfering information, which includes eyelids, eyelashes, and light [1]. An accurate iris segmentation method means that the iris region can include more discriminative features and get a higher final recognition accuracy [2]. For same-sensor iris segmentation, the database is compiled with the same acquisition equipment and shooting environment. These iris images in the training set and test set have similar data distribution, and thus most of the properly trained iris segmentation networks can achieve promising segmentation accuracy on the test set. For cross-sensor iris segmentation, uncorrelated noise (e.g., user cooperation, occlusion, illumination, gaze deviation, etc.) frequently appears in the cross-database situation, which can seriously threaten its segmentation performance [3]. Meanwhile, there is a significant imaging gap between training and testing sets in the cross-database scenario [4,5]. Therefore, cross-database iris segmentation is a challenging task [6].

Many traditional iris segmentation methods involved locating iris boundaries using variants of the Hough Transform [7,8] and the integro-differential operator [9,10]. These traditional iris segmentation algorithms can achieve satisfactory segmentation results on the ideal iris image. However, in most instances, iris images may contain a lot of irrelevant noise, and thus the segmentation accuracy of these algorithms will drop significantly.

In the past few years, CNNs have achieved milestones in image semantic segmentation. A series of CNNs-based semantic segmentation networks have constantly advanced state-of-the-art performance. At present, the existing iris segmentation methods are mostly based on several typical semantic segmentation networks, such as fully convolutional networks (FCN) [11], U-shaped net (U-Net) [12], Linknet [13], etc. Specifically, Jalilian et al. [14] first proposed an FCN-based iris segmentation network. Then, Chen et al. [15] proposed a DFCN combined with dense blocks to alleviate model overfitting and gradient vanishing. Mousumi Sardar et al. [16] proposed an interactive variant of UNet for iris segmentation, referred to as ISqEUNet. By introducing the squeeze and expand module, the model parameters are decreased by 48.39% compared with the original UNet. Tian et al. [17] proposed an iris segmentation algorithm SRN-UNet to solve the problem of low segmentation accuracy for segmenting low-quality iris images. In order to improve multi-source heterogeneous iris segmentation accuracy, Huo et al. [1] proposed a DMS-UNet based on DropBlock and shortcut branches. The DropBlock structure is used to improve the generalization ability of the network, and the shortcut branch is used to reduce the loss of information. The current iris segmentation networks have good segmentation accuracy on the same database. However, these networks have poor generalization ability and migration ability when different iris databases are used for training and test sets.

VIT [18] based on self-attention has the ability to extract global feature information because it performs self-attention computations on the entire image. However, the heavy computational cost hampers its application in semantic segmentation tasks. Liu et al. [19] proposed a hierarchical vision transformer based on shifted windows, referred to as a Swin T. Computational self-attention within a moving window can greatly reduce the computational cost while maintaining the global feature information ability. Compared with CNNs, Swin T pays more attention to global features but ignores the detailed features in the image. However, CNNs have better performance than Swin T in extracting image details such as texture features.

Our main innovations and contributions are as follows:

1. We proposed a bilateral segmentation backbone network that combines the benefits of Swin T with CNNs for accurate iris segmentation. Swin T is used to learn global and long-term semantic information interactions, and CNNs are used to extract fine-grained iris texture features and edge features
2. We designed a parallel structure based on dilated convolution to enhance the receptive field and capture rich iris feature information. MFIEM can extract multiscale context heterogeneous iris feature information.
3. In order to reduce the interference of irrelevant noise in the network, a channel attention mechanism module was used in this paper. CAMM can assign the importance of information on the channel, enhance the important features, suppress the useless features, and improve the representation ability of the network model.

## 2. Related Works

Most traditional iris segmentation algorithms need to prespecify that the iris region is a standard circle or ellipse. Meanwhile, there are also strict requirements (e.g., user cooperation, no obvious occlusion, etc.) for the collection environment. The methods based on deep neural networks can effectively compensate for the shortcomings of traditional methods. Therefore, deep learning technology and convolutional neural network have gradually become the mainstream methods to solve the iris segmentation task. Some CNNs-based iris segmentation methods are presented below.

Chen et al. [20] proposed a high-performance network architecture to improve the segmentation accuracy of low-quality iris images. Based on the encoding and decoding structure, the network introduces an improved skip connection structure to effectively fuse the spatial location information of low-level features and the semantic information of high-level features. Although the proposed method achieves promising segmentation accuracy, the network lacks sufficient training data.

The training data directly affects the performance of supervised iris segmentation networks. Although data augmentation techniques (e.g., scaling, flipping, cropping) have been successful in image classification tasks, these techniques are ineffective in the field of iris segmentation. To this end, Putri et al. [21] utilized generative adversarial networks to generate different types of iris images. The model generates a large number of iris images by using predefined iris masks and periocular masks. The model provides a new approach to solving small-sample iris image segmentation.

To improve the efficiency of the iris segmentation network, Miron et al. [22] designed a compact UNet network structure that requires only three down-sampling operations and three up-sampling operations. The network utilizes traditional convolutional layers and depth-wise separable convolutional layers to extract iris image features. Compared with UNet, the network can greatly reduce the training parameters while maintaining the segmentation accuracy.

In order to meet the demand for multiple scenarios iris segmentation, Huo et al. [23] proposed an Attention Mechanism UNet++ (AM-UNet++). They designed a deeply supervised learning scheme to train the network structure and used the pruning scheme to obtain four iris segmentation networks with different performances in the inference stage. AM-UNet++ (L1) and AM-UNet++ (L2) have more advantages in the number of parameters and computational cost, and thus they can be applied to low-performance devices or real-time devices. AM-UNet++ (L3) and AM-UNet++ (L4) have more advantages in segmentation accuracy, so they can be deployed in places with high security requirements. The pruning strategy is only adopted in the inference phase, and thus the network still requires a large number of computing resources and storage space capacity in the training phase.

Different from other segmentation networks, Wang et al. [3] proposed a unified multitask iris segmentation method. The network can generate not only iris segmentation masks but also parameterized inner and outer iris boundaries, which means that subsequent normalization operations can be implemented more easily. UNet is used as the backbone network of this method, and the iris mask, pupil mask, and iris outer boundary are selected as the input of the method. Pupil mask and iris outer boundary require manual annotation by experimenters, which undoubtedly increases the cost.

At present, most iris segmentation networks are trained and tested on the same database. Cross-database iris segmentation means that the segmentation network is trained on one database and tested directly on the other database. The cross-database iris segmentation requires that the segmentation network has a strong generalization ability, and thus it is a challenging task. Therefore, a bilateral segmentation backbone network based on MFIEM and CAMM is proposed in this paper.

## 3. Methods

**Figure 1:**
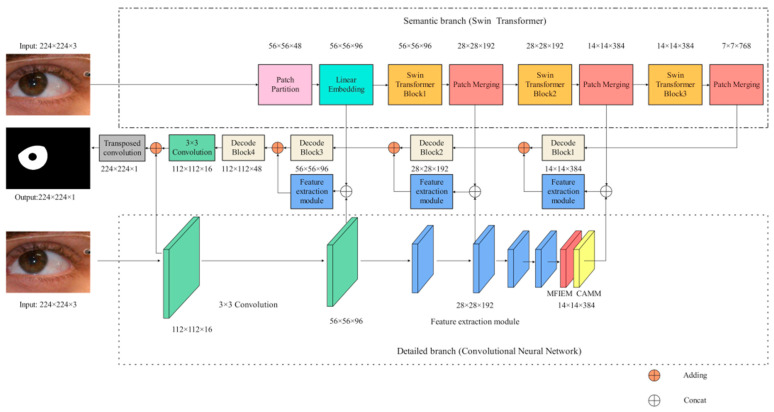
The iris segmentation network proposed in this paper (MFIEM: Multiscale feature information extraction module, CAMM: Channel attention mechanism module).

The framework of our network is illustrated in Figure 1. Specifically, the encoder consists of a semantic branch (Section 3.1) and a detailed branch (Section 3.2), which are used to extract iris image features. In the detailed branch, MFIEM (Section 3.3) is used to extract iris feature information at different scales, and CAMM (Section 3.4) is used to increase the feature weight of the iris region. The decoder is used to convert the iris feature information into iris semantic information. The implementation details of our structure are shown in Table 1. The size of the input image is 224 × 224 × 3 (H × W × C), where H and W represent the height and width of the feature map, respectively, and C represents the depth (the number of channels) of the feature map.

### 3.1. Design of the Semantic Branch

**Figure 2:**
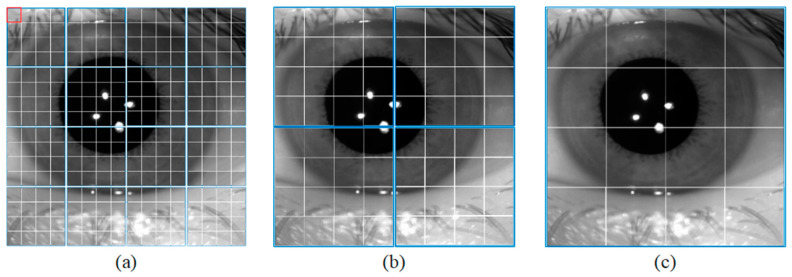
Window size for different blocks. (**a**) Swin Transformer Block1, (**b**) Swin Transformer Block2, and (**c**) Swin Transformer Block3.

The semantic branch consists of a patch partition layer, a linear embedding, three Swin T blocks, and three patch merging layers. Specifically, the patch partition layer is used to divide the input image into non-overlapping patches, and the linear embedding layer is used to adjust the number of channels. As shown in Figure 2a, the red rectangle represents a patch. The patch partition layer is implemented by a convolutional layer, which consists of 48 convolutional kernels with a kernel of size 4 × 4 and a stride of 4. Therefore, the width and height of the output feature map are reduced to 1/4 of the input feature map, and the number of channels is increased to 48. Then the feature map processed by linear embedding is sent into the Swin T blocks. The Swin T block is used to extract the feature information of the iris image, and the block does not change the size of the input feature map. The patch merging layer is used to perform down-sampling operations, which function similarly to the max-pooling operation in CNNs. This structure can reduce the resolution of the feature map and increase the number of channels of the feature map. Therefore, the width and height of the output feature map are reduced to 1/2 of the input feature map, and the number of channels is increased to two times that of the input feature map. The calculation process of the Swin T block is described in detail below.

The Swin T block performs the self-attentive computation in a local window. Self-attentive computation is computed as follows:

(1) $$Attention\left(Q,K,V\right)=Softmax\left(\frac{QK^{T}}{\sqrt{d}}+B\right)V,$$

where $Q,K,V\epsilon {\mathbb{R}}^{M^{2}\times d}$ denote the query, key, and value matrices, respectively. $M^{2}$ represents the number of patches in a window, d represents the dimension of query or key, and the bias matrix is denoted as *B*.

In the local window, each patch performs self-attentive computation with other patches to obtain global feature information. As shown in Figure 2a, the blue rectangle represents a window. Each window contains M × M patches. As the number of down-sampling increases, the size of the window also increases.

**Figure 3:**
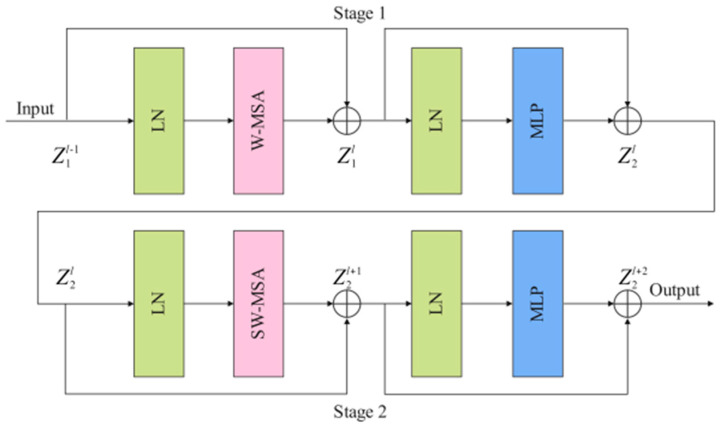
The implementation details of the Swin T block (LN: layer-norm, W-MSA: window-based multi-head self attention, SW-MSA: shifted window MSA, MLP: multi-head self-attention).

As shown in Figure 3, Swin T block consists of two stages: stage 1 and stage 2. Stage1 is composed of two layer-norm (LN) layers, a window-based multi-head self attention (W-MSA) module, two residual connections, and a multi-head self-attention (MLP) module. LN has a certain anti-overfitting effect, which makes the training process more stable. Shallow features and deep features are connected by residual connection. The calculation process of stage 1 is as follows:

(2) $$Z_{1}^{l}=W-MSA\left(LN\left(Z_{1}^{l-1}\right)\right)+Z_{1}^{l-1},$$

(3) $$Z_{2\;}^{l}=MLP\left(LN(Z_{1}^{l}\right))+Z_{1}^{l},$$

where $Z_{1}^{l}$ and $Z_{2}^{l}$ represent the outputs of the W-MSA module and the MLP module, respectively. The feature map $Z_{2}^{l}$ processed by stage 1 is sent to stage 2 as input data. W-MSA lacks effective information interaction between the windows, which limits its modeling power. To solve this problem, shifted window MSA (SW-MSA) is used in stage 2 instead of W-MSA to perform the self-attentive computation. The calculation process of stage 2 is as follows:

(4) $$Z_{2}^{l+1}=SW-MSA\left(LN(Z_{2}^{l}\right))+Z_{2}^{l},$$

(5) $$Z_{2}^{l+2}=MLP\left(LN\left(Z_{2}^{l+1}\right)\right)+Z_{2}^{l+1},$$

where $Z_{2}^{l+1}$ and $Z_{2}^{l+2}$ represent the outputs of the SW-MSA module and the MLP module, respectively. The feature map $Z_{2}^{l+2}\;$ processed by stage 2 is sent to the next Swin T block as input data.

### 3.2. Design of the Detailed Branch and Decoder Structure

**Figure 4:**
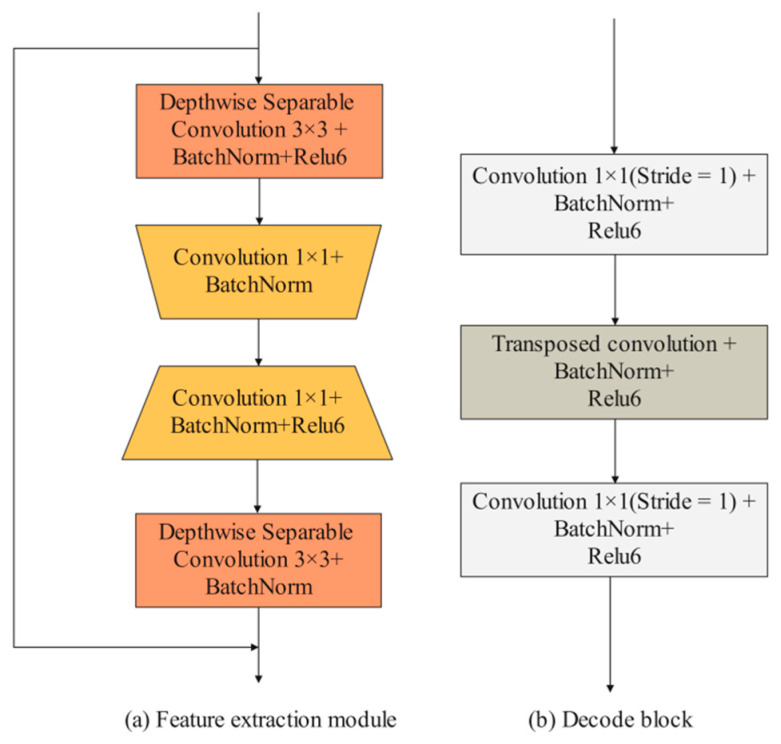
The design of feature extraction module and decode block.

Detailed branch: The input images are first convolved by two 3 × 3 convolution layers to extract iris features. Then, the feature maps pass through four feature extraction modules. Existing feature extraction modules mainly consist of some traditional convolutional layers (e.g., 3 × 3 convolution layers and 5 × 5 convolution layers) and depth-wise separable convolutional layers. In the iris segmentation task, using traditional convolutional layers, such as UNet, entails a lot of computational and hardware costs. However, using depth-wise separable convolutional layers can improve segmentation efficiency while maintaining segmentation accuracy. Compared with these two convolution methods, this paper designed an efficient feature extraction module. As shown in Figure 4a, the feature extraction module consists of two depth-wise separable convolution layers and two 1 × 1 convolution layers. It is worth noting that no activation function is added after the first 1 × 1 convolution and the second depth-wise separable convolution. When the input size and output size of the feature map are consistent, the input features and output features are fused through the shortcut branch structure. The first convolutional layer is used to reduce the number of channels of the feature map, and the second convolutional layer is used to increase the number of channels of the feature map. Using these 1 × 1 convolution layers to achieve dimensionality reduction and dimensionality generation operations can effectively encode channel information compared to using only depth-wise separation convolutions. MFIEM is inserted after the feature extraction module to extract multiscale iris feature information, and the CAMM is inserted at the end of the detailed branch.

Decoder structure: The feature map is first passed through four decoder blocks to extract features. As illustrated in Figure 4b, the decode block consists of two 1 × 1 convolution layers and a transposed convolution layer. The transposed convolution layer is used to expand the length and width of the input feature map. Finally, the 3 × 3 convolution layer is used to adjust the dimension of the feature map, and the transposed convolution layer is used for the final prediction.

### 3.3. Multiscale Feature Information Extraction Module

**Figure 5:**
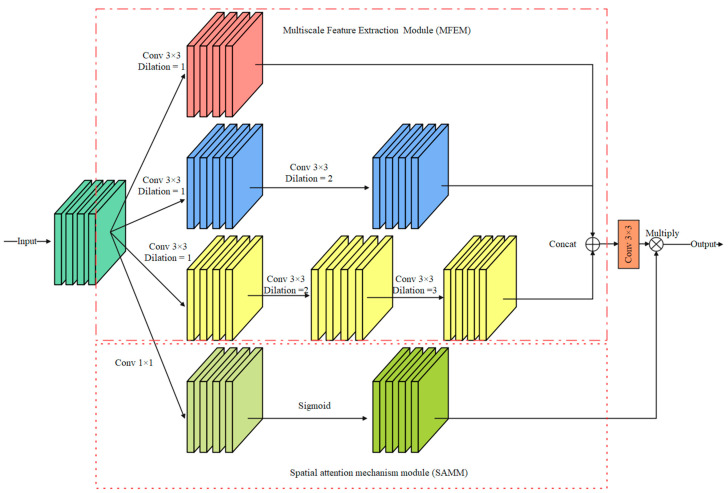
Multiscale feature information extraction module (MFIEM).

In the detailed branch, small convolution kernels, such as 3 × 3 convolution and 5 × 5 convolution, can effectively extract the edge detail information of the iris image but ignore the spatial correlation of the image. Inspired by the Atrous Spatial Pyramid Pooling [24] (ASPP) module, a multiscale feature information extraction module is proposed in this paper. As shown in Figure 5, the module consists of a multiscale feature extraction module (MFEM) and a spatial attention mechanism module (SAMM).

Multiscale feature extraction module: Dilated convolution (DC) controls the receptive field of the convolution kernel by setting different dilation rates. Using the DC does not add extra model parameters and computation costs. Therefore, the proposed MFEM employs dilated convolution with different DC to extract features, and the dilation rates are set to {1, 2, 3}. The output feature maps are stacked in the dimension of the channel, and a 3 × 3 convolution layer is used to adjust the dimension of the feature maps.

Spatial attention mechanism module: The module consists of a 1 × 1 convolution layer and a Sigmoid function. Then, a 1 × 1 convolution layer is used to capture global feature information. The output feature map is multiplied by the feature map processed by the 3 × 3 convolution layer in the spatial dimension to achieve information calibration.

Compared with traditional convolution, using convolution kernels with different receptive fields can obtain more accurate and comprehensive feature information. SAMM is lightweight because it only uses a 1 × 1 convolution layer.

### 3.4. Channel Attention Mechanism Module

**Figure 6:**
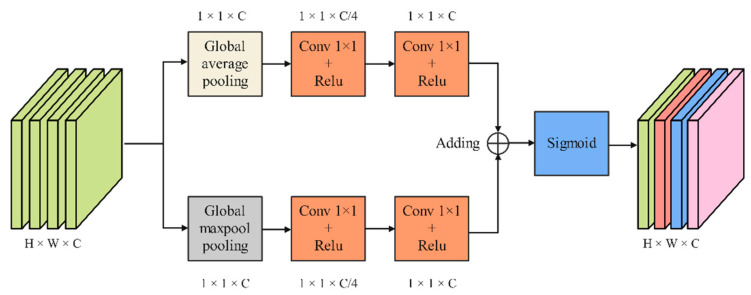
Channel attention mechanism module.

As shown in Figure 6, CAMM extracts features through two branches, which consist of a pooling layer and two 1 × 1 convolution layers. First, the input feature maps pass through a global max pooling layer based on the width direction and a global average pooling layer based on the height direction, respectively. Then, the output feature maps are sent to the first 1 × 1 convolution layer to compress the channel. The second 1 × 1 convolution layer is used to learn the weights for each channel. The feature maps output by the two branches is fused by adding operations. Finally, feature maps redistribute the weights on the channels through the Sigmoid function.

## 4. Experimental Configurations

### 4.1. The Iris Image Database

**Figure 7:**
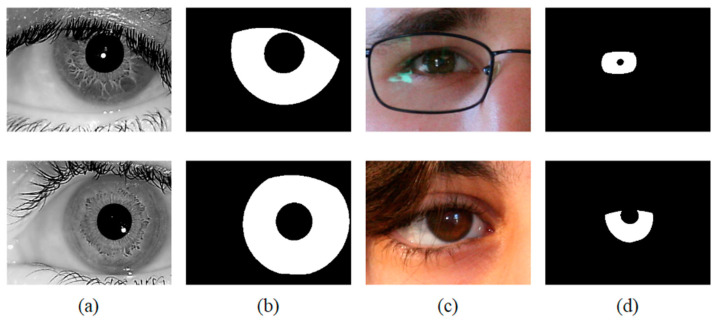
Image samples from the adopted iris databases (**a**) IITD, (**b**) Ground-truth masks of IITD, (**c**) UBIRIS.v2, (**d**) Ground-truth masks of UBIRIS.v2.

The databases used for the comparison experiment: IITD iris database [25] is provided by the IIT Delhi, New Delhi. UBIRIS.v2 iris database [26] is proposed by the University of Beira Interior. The detailed parameters of these datasets are listed in Table 2, and some image samples are shown in Figure 7. The iris databases are divided into three parts, training set, validation set, and test set, which are split according to a ratio of 7:1:2. This pattern of dividing the training and test sets is used by most iris segmentation methods [1,15,27,28]. For the IITD iris database, 1580 iris images are used to train the segmentation network, 220 iris images are used to adjust network weights, and 440 iris images are used to measure the accuracy of the segmentation network. For the UBIRIS.v2 iris database, 1575 iris images are used to train the segmentation network, 225 iris images are used to adjust network weights, and 450 iris images are used to measure the accuracy of the segmentation network. In order to fairly evaluate the segmentation accuracy of different methods, the training and testing sets of the two iris databases used in our experiment are the same as those of DMS-UNet [1] and Linknet [13]. For other segmentation networks, the number of training and test sets used in this article is very close to the number of training and test sets used in other respected studies. Therefore, the experimental results in this paper are reliable.

**Figure 8:**
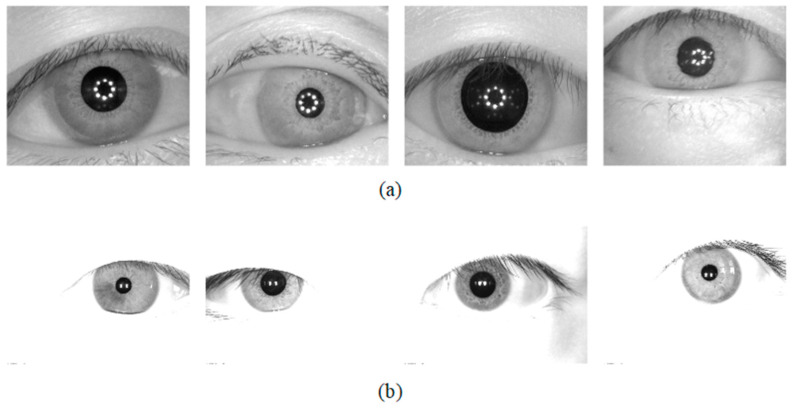
Image samples from the adopted iris databases (**a**) CASIA-v4.0 and (**b**) JLU-4.0.

The database used for the Universality experiment: The segmentation method should be tested on the database which is not used in the training stage. To this end, two iris databases were chosen for the generality experiments of the network. The CASIA-v4.0iris database is captured with a self-developed close-up iris camera [29]. The JLU-4.0 [30] iris database is captured by an iris collector independently developed by Jilin University. CASIA-v4.0 and JLU-4.0 were obtained under near-infrared illumination, and some image samples are shown in Figure 8.

### 4.2. Metrices Used in the Evaluation Section and Experimental Implementation

We measure the segmentation accuracy of the network by the following evaluation metrics. Specifically, mean intersection over union (MIOU), f1 score (F1), and error score2 (NICE2) [31] are used to evaluate the segmentation accuracy. The value of MIOU, F1, and NICE2 is between zero and one. The closer the MIOU value and F1 value are to 0, the worse the performance of the segmented network. The closer the MIOU value and F1 value are to 1, the better the performance of the segmented network. However, the closer the value of NICE is to 1, the worse the performance of the network. The closer the value of NICE is to 0, the better the performance of the network.

(6) $$\mathrm{MIOU}\;=\frac{1}{n}\sum_{n}^{i=1}{\left[\frac{\mathrm{TP}}{\mathrm{FP}+\mathrm{FN}+\mathrm{TP}}\right]}_{i}$$

(7) $$F1=\frac{2\mathrm{TP}}{2\mathrm{TP}+\mathrm{FP}+\mathrm{FN}}$$

(8) $$\mathrm{NICE}2=\frac{1}{2}\left(\frac{\mathrm{FN}}{\mathrm{FN}+\mathrm{TP}}+\frac{\mathrm{FP}}{\mathrm{FP}+\mathrm{TN}}\right)$$

The hardware platform is a single NVIDIA GeForce RTX 3090 GPU with 24 GB of memory. We implement our method based on Pytorch (version 1.7.1). The Dice function [32] is adopted as the loss function in this paper. The optimization is performed by using the Adam optimizer with an initial learning rate that equals 0.001. The proposed model is trained for 50 epochs with a batch size of 32.

## 5. Experimental Results

### 5.1. Ablation Experiments

Table 3 summarizes the ablation results with different feature extraction networks. Using only CNNs achieves better iris segmentation accuracy than using only Swin T. Compared with using CNNs, Swin T can extract the global features of the image, which will cause the network to lose some detailed feature information in the encoder stage.

It is seen from Table 3 that using both CNNs and Swin T achieves the best iris segmentation. Specifically, for the IITD database, compared with using only a single branch network, the MIOU of our network is improved by 0.83% and 0.43%, respectively. At the same time, for the UBIRIS.v2 database, the MIOU of our network is improved by 1.21% and 0.76%, respectively. Using a dual-branch network can simultaneously extract global and local features, which helps to improve the segmentation precision of our network.

**Figure 9:**
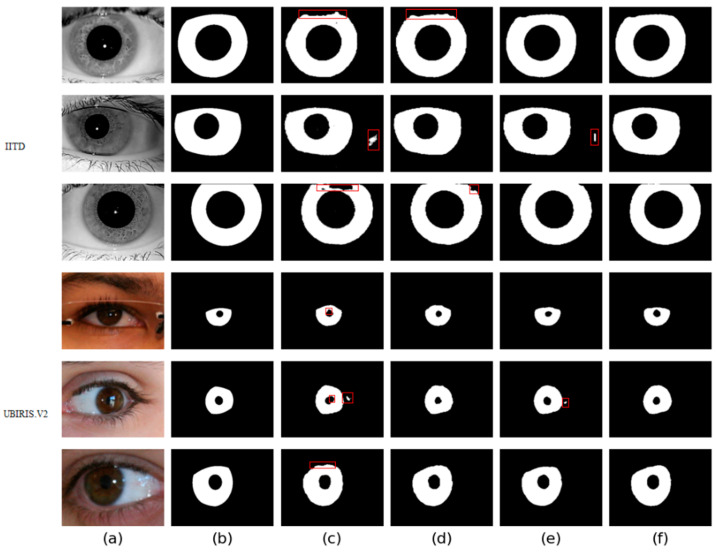
Segmentation results of different networks on two databases: (**a**) Original image, (**b**) Ground truth, (**c**) Results of baseline network, (**d**) Results of baseline network based on CAMM, (**e**) Results of baseline network based on MFIEM, and (**f**) Results of the proposed network.

Four different networks are designed for the ablation study. Swin T and CNNs are used as the backbone of the benchmark network to extract iris image features. Firstly, the benchmark network does not use MFIEM and CAMM. Then, two different models use MFIEM and CAMM, respectively. Finally, we use the proposed network as the fourth network. Figure 9 shows the segmentation results of different networks. The third column shows the results of the baseline network, the fourth column is the results of the baseline network after adding the CAMM, and the fifth column is the results of the baseline network after adding the MFIEM.

As shown in Table 4, the MIOU values of the proposed network on the two databases are 0.9694 and 0.9566, respectively, which are 0.88% and 0.81% higher than the baseline network. The segmentation results of the baseline network contain some misclassifications. Compared with the baseline network, our network achieves better segmentation results. Therefore, it is effective to use MFIEM and CAMM in the iris segmentation task.

The MIOU values of the network with the MFIEM on two iris databases are respectively 0.58% and 0.43% higher than the baseline network. For the benchmark network, some iris areas are segmented into the background area, and some pupil areas are under-segmented. Using the MFIEM can effectively capture multiscale feature information, which is critical for identifying small target areas.

As illustrated in Table 4, the MIOU of the baseline network based on CAMM is improved by 0.43% and 0.41% on two iris databases, respectively. The misclassified pixels of the benchmark network based on CAMM are greatly reduced. Adding the CAMM can effectively reduce the network’s response to irrelevant noise.

### 5.2. Comparison with Conventional Segmentation Networks

Compared with other conventional algorithms, as shown in Table 5, our iris segmentation network achieves higher MIOU and F1 and lower NICE on two iris databases, with MIOU reaching 0.9694 and 0.9566, and F1 reaching 0.9844 and 0.9774 on the iris datasets of IITD and UBIRIS.V2, respectively. For UBIRIS.v2, conventional iris segmentation algorithms cannot accurately segment the iris region because iris images contain a lot of irrelevant noise. Compared with Ahmad’s method, the F1 of our network is improved by 3.4% on the IITD iris database. Compared with the Ifpp algorithm, the NICE2 of our network is improved by 95.06% on the UBIRISv2 iris database.

### 5.3. Comparison with Algorithms Based on CNNs

**Figure 10:**
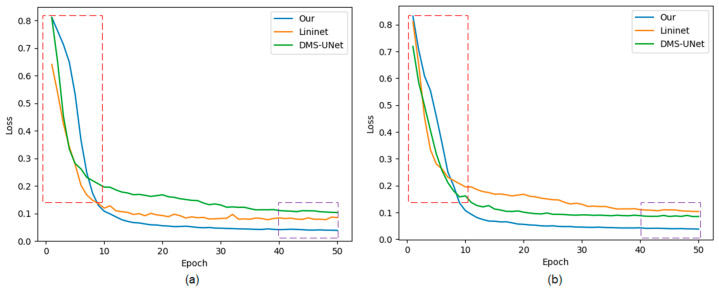
The loss curves of the proposed network, Linknet, and DMS-UNet on two databases: (**a**) IITD and (**b**) UBIRIS.V2. Red frame: the early stage of network training; purple frame: the later stage of network training.

The approaches labeled with the symbol “*” represent our implementation of the algorithm. The method that is not marked with this symbol represents the experimental data from respected studies. Figure 10 shows the training loss curves of different segmentation networks on the training set. Early phases of training are marked with a red frame, and late stages of training are marked with a purple frame.

The convergence speed of our network is slower than that of the network based on a CNN. There is a certain semantic gap between global features captured by Swin T and local features captured by CNN, which causes the network to learn slowly before 10 epochs.

Our method achieves lower loss values compared with other segmentation networks. Meanwhile, the loss curves of our network converge rapidly with increasing epochs, and the curves are stable without significant oscillations, indicating that the network has more fully learned than other segmentation methods.

As shown in Table 6, it can be observed that UNet outperforms other semantic segmentation networks across all metrics on the UBIRIS.v2 database. DeepLabV3 and Linknet are proposed to segment the universal dataset (e.g., PASCAL VOC 2012), while UNet is proposed to segment the dataset for medical images. Iris image segmentation can be regarded as a sub-task of medical image segmentation, and thus UNet has achieved promising performance in the field of iris image segmentation. This is why most iris segmentation methods use UNet as the baseline network. DMS-UNet uses the DropBlock structure to enhance the network in terms of learning more useful iris features. Therefore, DMS-UNet achieves higher MIOU and F1 and lower NICE2 than UNet on the UBIRIS.v2 test set. Compared with the other segmentation networks, our method can achieve better segmentation accuracy. Specifically, on the IITD test set, our network gets the highest MIOU of 0.9694, the highest F1 of 0.9844, and the lowest NICE2 of 0.016. Compared with DMS-UNet, the MIOU and F1 of our network are improved by 0.95% and 0.48%. Meanwhile, our segmentation network gets the highest MIOU and F1 of 0.9566 and 0.9774 on the UBIRIS.v2 database and the lowest NICE2 of 0.0196 on the UBIRIS.v2 database. Compared with segmentation network Linknet, the MIOU and F1 of our network are improved by 4.03% and 2.16%, respectively. Our network surpasses the DMS-UNet, the latest high-performance heterogeneous iris segmentation method, by about 0.97%, 0.5%, and 2.09% on MIOU, F1, and NICE2, respectively. DMS-UNet and Linknet use depthwise separable convolution and traditional 3 × 3 convolution to extract iris image features, respectively. These methods cannot learn global and long-term semantic information interactions well.

**Figure 11:**
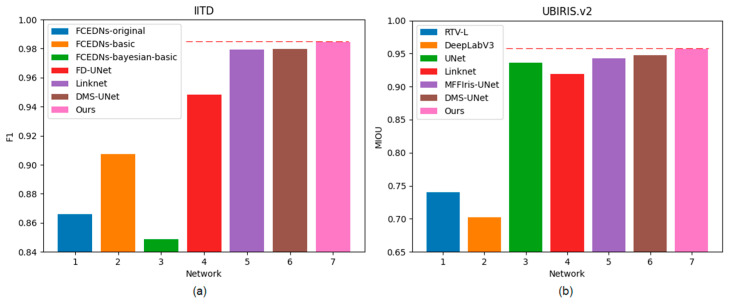
Histogram of F1 and MIOU on two databases: (**a**) IITD and (**b**) UBIRIS.V2.

Our bilateral segmentation backbone network can not only extract the global feature information but also extract the detailed features. Therefore, the proposed segmentation network can achieve an outstanding segmentation effect on different iris databases. Figure 11 shows the comparison of different methods on two databases.

### 5.4. The Segmentation Results of Different Databases

For iris images occluded by eyelashes, it is difficult for Linknet and DMS-UNet to accurately predict the iris boundary. However, adding the CAMM module to the network can largely address this deficiency, and thus our method can accurately segment the iris region.

**Figure 12:**
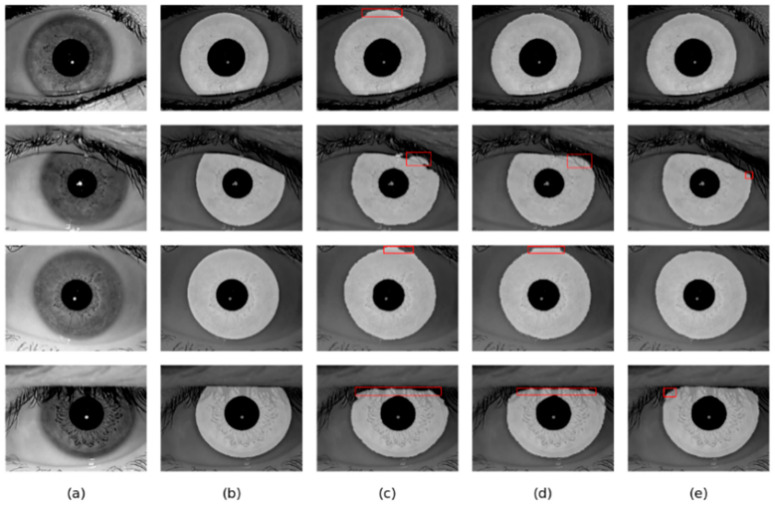
Segmentation results of different methods on the IITD database: (**a**) Original image, (**b**) Ground truth, (**c**) results of Linknet, (**d**) results of DMS-UNet, and (**e**) results of the proposed network.

**Figure 13:**
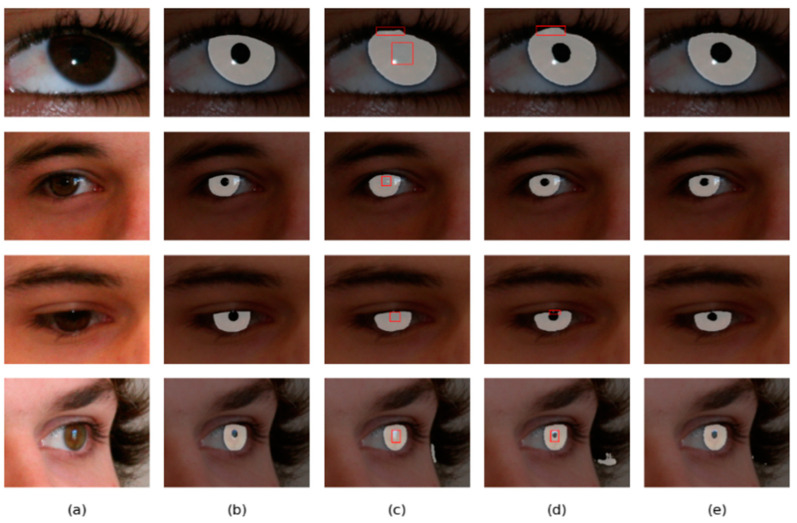
Segmentation results of different methods on the UBIRIS.v2 database: (**a**) Original image, (**b**) Ground truth, (**c**) results of Linknet, (**d**) results of DMS-UNet, and (**e**) results of the proposed network.

As shown in Figure 12 and Figure 13, the overall result of DMS-UNet is good, but the iris edge fitting is not good enough. Linknet cannot accurately segment some small regions, such as the pupil. However, the segmentation results of our network are closer to the real label graph. The proposed network can calculate the relationship between elements in a wide range, which is beneficial to obtain the global receptive field of small target areas. Therefore, our network can accurately segment the pupil area.

### 5.5. The Universality of Network Experiment

**Figure 14:**
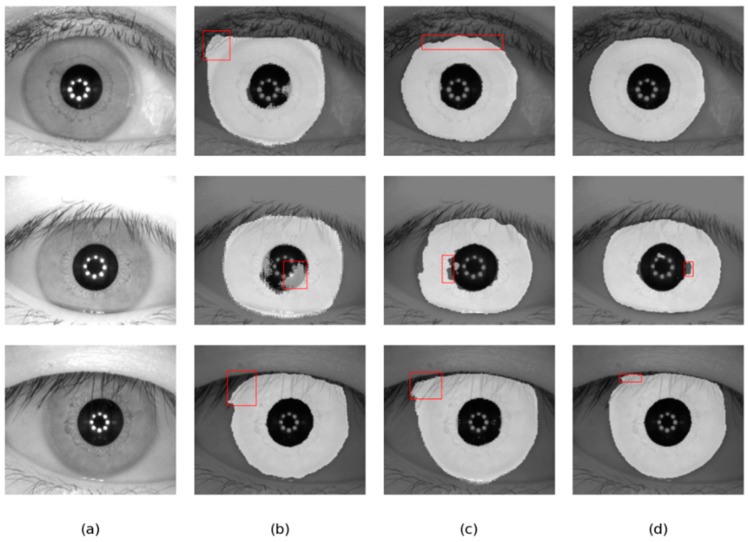
Segmentation results of different methods on the CASIA-V4.0 database: (**a**) Original image, (**b**) results of Linknet, (**c**) results of DMS-UNet, and (**d**) results of the proposed network.

**Figure 15:**
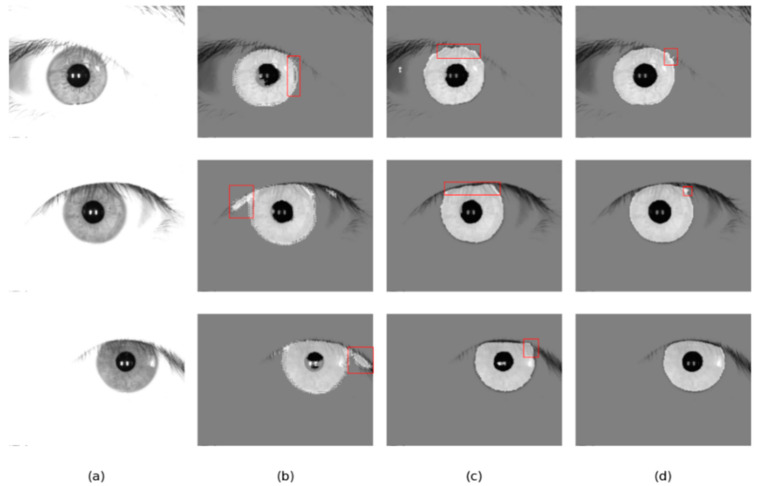
Segmentation results of different methods on the JLU-4.0 database: (**a**) Original image, (**b**) results of Linknet, (**c**) results of DMS-UNet, and (**d**) results of the proposed network.

To verify the generality and practicality of the iris segmentation network, our network, Linknet, and DMS-UNet are trained on the IITD database and tested on the CASIA-V4.0 (Figure 14) and JLU-4.0 (Figure 15) databases. Since the JLU-4.0 iris database does not provide corresponding ground-truth masks, we only conduct qualitative analysis based on the segmentation results of different networks.

As shown in Table 7, our segmentation network has significant advantages under the cross-database protocol. Compared with Linknet and DMS-UNet, our iris segmentation network achieves higher MIOU and F1 and lower NICE2 on the IITD test set, with MIOU reaching 0.9425 and F1 reaching 0.9701. In order to further explore the reason for this, we randomly selected some iris images segmented by different networks for qualitative analysis.

As shown in Figure 14 and Figure 15, the results of DMS-UNet and Linknet are unsatisfactory, which indicates that these networks have poor generalization and migration ability. Specifically, for the region with eyebrows and eyelashes in the image, the segmentation result of Linknet contains some misclassification of the iris region pixels for these irrelevant noise pixels. For shaded regions in the iris image, DMS-UNet incorrectly identifies the shadow area as the iris area (e.g., the first row of Figure 15). However, compared with other networks, the segmentation results of our network have fewer pixel misjudgments.

Based on the above segmentation results, the proposed network gets better segmentation accuracy than other segmentation networks on a database that is not used in the training stage. This shows that our network has learned the real iris features and has universality for iris images taken under different conditions. Therefore, the network has a certain application value.

## 6. Conclusions

To accurately segment multisource heterogeneous iris images, we proposed an architecture based on a bilateral segmentation backbone network. This bilateral network can combine the advantages of Swin T and CNNs. The semantic branch based on Swin T is used to extract the global feature information of images, and the detailed branch based on CNNs is used to extract the detailed features.

The ablation experiment and visualization results demonstrate that using the MFIEM module can efficiently extract spatial contextual information from the iris images. Using CAMM gives more importance to iris regions and ignores irrelevant ones. Our network can achieve SOTA performance. The universality experimental results show that the network has a certain migration and generalization ability. Therefore, our method allows users to choose different acquisition devices to flexibly form their own iris recognition system.

## Figures and Tables

**Table 1:** Network structure details.

Structure	Input Size (H × W × C)	Operation	Stride	Output Size (H × W × C)
Semantic branch	224 × 224 × 3	Patch Partition	4	56 × 56 × 48
	56 × 56 × 48	Linear Embedding	1	56 × 56 × 96
	56 × 56 × 96	Swin T Block1	1	56 × 56 × 96
	56 × 56 × 96	Patch Merging	2	28 × 28 × 192
	28 × 28 × 192	Swin T Block2	1	28 × 28 × 192
	28 × 28 × 192	Patch Merging	2	14 × 14 × 384
	14 × 14 × 384	Swin T Block3	1	14 × 14 × 384
	14 × 14 × 384	Patch Merging	2	7 × 7 × 768
Detailed branch	224 × 224 × 3	3 × 3 Convolution	2	112 × 112 × 16
	112 × 112 × 16	3 × 3 Convolution	2	56 × 56 × 96
	56 × 56 × 96	Feature extraction module	2	28 × 28 × 192
	28 × 28 × 192	Feature extraction module	1	28 × 28 × 192
	28 × 28 × 192	Feature extraction module	2	14 × 14 × 384
	14 × 14 × 384	Feature extraction module	1	14 × 14 × 384
	14 × 14 × 384	MFIEM	1	14 × 14 × 384
	14 × 14 × 384	CAMM	1	14 × 14 × 384
Decoder	7 × 7 × 768	Decode Block1	2	14 × 14 × 384
	14 × 14 × 384	Decode Block2	2	28 × 28 × 192
	28 × 28 × 192	Decode Block3	2	56 × 56 × 96
	56 × 56 × 96	Decode Block4	2	112 × 112 × 48
	112 × 112 × 48	3 × 3 Convolution	1	112 × 112 × 16
	112 × 112 × 16	Transposed convolution	2	224 × 224 × 1

**Table 2:** The characteristics of iris image databases.

Property	IITD	UBIRIS.v2
Image Size	320 × 240	400 × 300
Input Size	224 × 224	224 × 224
The number of training sets	1580	1575
The number of validating sets	220	225
The number of testing sets	440	450
Modality	near-infrared	visible light
Color	gray-level	RGB

**Table 3:** Results of network ablation experiments.

Database	Network	MIOU	F1	NICE2
IITD	Swin T	0.9530	0.9758	0.0274
	CNNs	0.9568	0.9779	0.0214
	Swin T + CNNs (Ours)	**0.9609**	**0.9800**	**0.0212**
UBIRIS.v2	Swin T	0.9376	0.9670	0.0316
	CNNs	0.9417	0.9693	0.0303
	Swin T + CNNs (Ours)	**0.9489**	**0.9738**	**0.0226**

Note: Bold values represent the best iris segmentation accuracy in the comparison methods.

**Table 4:** Network ablation experiments.

Database	Network	MIOU	F1	NICE2
IITD	Baseline	0.9609	0.9800	0.0212
	Baseline + MFIEM	0.9665	0.9829	0.0180
	Baseline + CAMM	0.9650	0.9822	0.0182
	Ours	**0.9694**	**0.9844**	**0.0160**
UBIRIS.v2	Baseline	0.9489	0.9738	0.0226
	Baseline + MFIEM	0.9544	0.9763	0.0202
	Baseline + CAMM	0.9528	0.9754	0.0216
	Ours	**0.9566**	**0.9774**	**0.0196**

Note: Bold values represent the best iris segmentation accuracy in the comparison methods.

**Table 5:** Comparison with conventional algorithms on two iris databases.

Database	Approach	MIOU	F1	NICE2
IITD	Ahmad [33]	-	0.9520	-
	GST [34]	-	0.3393	-
	Ours	**0.9694**	**0.9844**	**0.0160**
UBIRIS.v2	Chat [35]	-	0.1048	0.4809
	Ifpp [36]	-	0.2899	0.3970
	Wahet [37]	-	0.1977	0.4498
	Osiris [38]	-	0.1865	-
	IFPP [39]	-	0.2852	-
	Ours	**0.9566**	**0.9774**	**0.0196**

Note: Bold values represent the best iris segmentation accuracy in the comparison methods.

**Table 6:** Comparison with algorithms based on CNNs on two iris databases.

Database	Approach	MIOU	F1	NICE2
IITD	FCEDNs-original [14]	-	0.8661	0.0588
	FCEDNs-basic [14]	-	0.9072	0.0438
	FCEDNs-Bayesian-basic [14]	-	0.8489	0.0701
	FD-UNet [27]	-	0.9481	0.0258
	Linknet [13] *	0.9595	0.9793	0.0188
	DMS-UNet [1] *	0.9603	0.9797	0.0176
	Ours	**0.9694**	**0.9844**	**0.0160**
UBIRIS.v2	FCEDNs-original [14]	-	0.7691	0.1249
	FCEDNs-basic [14]	-	0.7700	0.1517
	FCEDNs-Bayesian-basic [14]	-	0.8407	0.1116
	RTV-L [28]	0.7401	0.8597	-
	DeepLabV3 [28]	0.7024	0.8755	-
	UNet [40]	0.9362	0.9553	-
	DFCN [15]	-	0.9606	0.0204
	Linknet [13] *	0.9195	0.9567	0.0316
	MFFIris-UNet [28]	0.9428	0.9659	-
	DMS-UNet [1] *	0.9474	0.9725	0.0248
	Ours	**0.9566**	**0.9774**	**0.0196**

* The symbol represents our implementation of the algorithm. Note: Bold values represent the best iris segmentation accuracy in the comparison methods.

**Table 7:** Comparison with other networks on the CASIA-V4.0 database.

Approach	MIOU	F1	NICE2
Linknet [13] *	0.9096	0.9520	0.0538
DMS-UNet [1] *	0.8826	0.9369	0.0434
Ours	**0.9425**	**0.9701**	**0.0337**

* The symbol represents our implementation of the algorithm. Note: Bold values represent the best iris segmentation accuracy in the comparison methods.

## Data Availability

The research in this paper uses the IITD iris image database provided by IIT Delhi, New Delhi, India; UBIRIS.v2 iris image database provided by the Department of Computer Science, University of Beira Interior; CASIA iris image database provided by the Chinese Academy of Science; JLU iris image database provided by Jilin University. IITD iris image database can be found in [25] and the UBIRIS.v2 iris database can be found in [26]. CASIA-v4 iris database can be found here: http://www.cbsr.ia.ac.cn/china/Iris%20Databases%20CH.asp (accessed on 7 September 2020). JLU-4.0 iris database can be found here: http://www.jlucomputer.com/index/irislibrary/irislibrary.html (accessed on 7 September 2020).
